# Rare Conditions of Hyperandrogenism Through Lifespan: A Case Series

**DOI:** 10.1155/crie/8604843

**Published:** 2025-11-27

**Authors:** Maria Apostolopoulou, Robert Taayedi, Cosmin Paul Sărac, Frank Demtröder

**Affiliations:** ^1^Zentrum für Endokrinologie, Diabetologie, Rheumatologie, ÜBAG MVZ Dr. Eberhard und Partner und Klinikum Dortmund, Dortmund, Germany; ^2^Department of Internal Medicine-Endocrinology, University Hospital of Larissa, Faculty of Medicine, University of Thessaly, Larissa, Greece; ^3^Department of Gynecology and Obstetrics, Christliches Klinikum Unna, Unna, Germany

## Abstract

A wide spectrum of clinical entities can lead to pre and postmenopausal hyperandrogenism, which is characterized by slow or more rapid onset of virilizing symptoms (menstrual irregularities, hirsutism, androgenetic alopecia). Functional hyperandrogenism in the context of polycystic ovary syndrome (PCOS) remains the most prevalent cause for hyperandrogenism both in pre and postmenopausal females; however, other clinical entities such as ovarian hyperthecosis and benign or malignant neoplasms (e.g., adrenal androgen-secreting adenomas and ovarian tumors of androgen-secreting cells) are often challenging to diagnose. Laboratory testing should include measurement of testosterone, sex hormone binding globulin (SHBG), gonadotropins, estradiol, androstenedione, dehydroepiandrosterone sulfate (DHEA-S), and 17-OH-progesterone values, as well as markers of other endocrine disorders leading to secondary hyperandrogenism, especially Cushing's syndrome. Testosterone values of more than 150 ng/dL generally require further investigation, and increased DHEA-S (more than 700 μg/dL) is suggestive of adrenal androgen-secreting tumors. Androgen suppression during prolonged dexamethasone test can facilitate differential diagnosis between adrenal and ovarian androgen excess production and point to autonomous production in case of tumors. In case of smaller ovarian tumors (e.g., Leydig cell), imaging might not be diagnostic, so that in case of high clinical suspicion, selective ovarian catheterization can be a valuable tool, when available. In this paper, we highlight four rare conditions of hyperandrogenism beyond PCOS, each reflecting specific stages or challenges across the female lifespan. We suggest that detailed biochemical testing and high clinical suspicion should promptly lead to valuable invasive diagnostic tools (ovarian catheterization/laparoscopy) in case imaging is not diagnostic.

## 1. Introduction

Clinical or biochemical hyperandrogenism in females is one of the most frequent reasons for consultation in endocrinology practice. The most prevalent cause of hyperandrogenism in pre and postmenopausal females remains polycystic ovary syndrome (PCOS), affecting about 10% of female population of reproductive age, and its persistence during menopause [1, 2]. Differential diagnosis includes late-onset congenital adrenal hyperplasia (CAH), ovarian hyperthecosis, followed by more rare causes, such as ovarian and even less frequently adrenal androgen-secreting tumors [1, 3]. Excessive cortisol production and more specifically ACTH-dependent Cushing's is also related to increased adrenal androgen production, and in 70%–80% of cases, presents with menstrual irregularities and hirsutism in females [4]. Besides their effects on fertility and action on hair follicles, hyperandrogenism is associated with increased risk for diabetes mellitus, insulin resistance, obesity, hypertension, and cardiovascular mortality [5], so that early diagnosis and treatment should be warranted.

Clinical examination and laboratory testing remains the main tool to differentiate adrenal from ovarian androgen production and imaging is further needed to confirm diagnosis in cases an underlying tumor is suspected due to the level of androgens, usually a total testosterone above 5 nmol/L (~150 ng/dL) or a dehydroepiandrosterone sulfate (DHEA-S) above 20 µmol/L (~700 μg/dL) in premenopausal women are proposed to reflect such a condition [6]. DHEA-S represents exclusively adrenal androgen secretion, and solely testosterone-secreting tumors are extremely rare [7]. When a transvaginal ultrasound fails to identify an ovarian tumor, MRI imaging may be helpful [8], whereas CT and MRI are suitable to detect adrenal tumors. Selective ovarian vein/adrenal catheterization can be useful if imaging fails to localize the origin of a biochemically proven tumor, especially in cases where fertility needs to be preserved [9].

However, in some cases, diagnosis may be challenging because of coexisting underlying endocrinopathies (e.g., functional hyperandrogenism in the context of obesity/PCOS, CAH) or negative initial imaging, so that high clinical suspicion, taking into consideration the onset and severity of androgenetic effects as well as the magnitude of androgen elevation, should prompt physicians to further examinations. In this case series, we present four nonconsecutive cases of female hyperandrogenism, which were selected due to challenging diagnosis and presence of concomitant conditions (mild autonomous cortisol secretion [MACS], functional hyperandrogenism, ovarian borderline tumor, hormone replacement therapy), and we report our approach from diagnosis to post-treatment management. All laboratory measurements were performed in the morning (08:00–10:00 a.m.). In premenopausal females, hyperandrogenism was confirmed in the early follicular phase of menstrual cycle. In the first case, we report on a postmenopausal woman presenting with adrenal adenoma and cushingoid features with persistent hyperandrogenism after unilateral adrenalectomy and negative imaging of her ovaries, who was finally diagnosed with ovarian hyperthecosis. Cases 2 and 3 show one post and one premenopausal female with known PCOS and diagnosis of unilateral ovarian androgen-secreting tumor, and in case 4, we present a patient with a very rare androgen-secreting adrenal adenoma, with persistent mild functional hyperandrogenism following adrenalectomy.

## 2. Case 1

**Figure 1:**
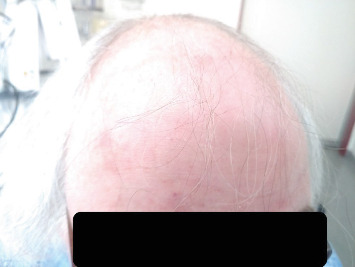
Androgenetic alopecia (grade III in Ludwig scale) in the patient with ovarian hyperthecosis (case 1).

**Figure 2:**
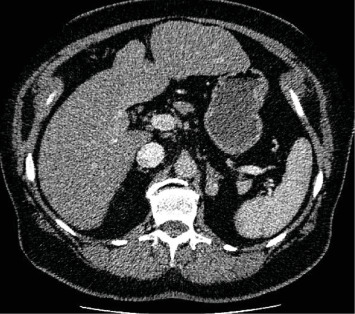
Abdominal CT scan demonstrating the adrenal adenoma of the left adrenal gland in a patient, with mild autonomous cortisol production and related comorbidities (case 1).

At first, we present the case of a 58-year-old postmenopausal woman, who presented in our endocrinology center for a preoperative endocrinological check-up before planned adrenalectomy due to an adrenal adenoma and autonomous cortisol secretion (ACS). Clinical examination revealed central adiposity (BMI 33,6 kg/m^2^, waist circumference: 122 cm) and increased blood pressure (160/98 mmHg). Furthermore, androgenetic alopecia (grade III on Ludwig scale, Figure 1), hirsutism, and striae rubriae were present. Ludwig scale scoring ranges from I to III and is used to describe hair loss patterns in females [10]. According to patient's history, frontotemporal hair loss was present since the last 2 years, and the patient was using a wig. This hair loss was initially attributed to methotrexate therapy, which she received to treat psoriasis, and was not further examined. The patient reported rapid development of type 2 diabetes in the last 2 months with an HbA1c of 12%, which was treated with a combination of metformin, sitagliptin, and basal insulin. Due to rapid diabetes development, her family doctor performed an overnight 1 mg dexamethasone suppression test, which showed a MACS (2.2 mcg/dL). In order to minimize the possibility of false-positive testing due to obesity, a 2 mg dexamethasone test was performed [11], and showed an identical result. Test repetition in our practice was offered but rejected by the patient due to distance and time limitations. Preoperative testing in our center showed a pronounced isolated testosterone increase, without increases in androstenedione or DHEA-S (Table 1). Morning ACTH (10 am) was suppressed, 24-h urine cortisol and cortisol saliva test did not detect any abnormalities. CT- imaging revealed a smoothly marginated, oval lesion located in the left adrenal gland with a maximal diameter of 2.2 cm × 1.5 cm (Figure 2). Abdominal MRI imaging was also performed, and the lesion demonstrated intermediate signal intensity on both T1- and T2-weighted images, without evidence of regressive calcifications, intralesional hemorrhage, or significant diffusion restriction. Following contrast administration, the lesion showed moderate contrast enhancement with subsequent washout over time, suggestive of an adrenal adenoma. The patient was referred for adrenalectomy due to MACS with related comorbidities. Primary hyperaldosteronism and pheochromocytoma were excluded by a normal aldosterone/renin-ratio and plasma (nor)-metanephrine measurements, respectively. Before surgery, the patient was referred to her gynecologist for a transvaginal ultrasound, which was not diagnostic for ovarian processes.

Adrenalectomy was successfully performed; histology revealed multinodular adrenal hyperplasia without suspicion of malignancy. HbA1c was substantially improved after surgery. Postoperative testing showed unaltered testosterone levels, so the patient was further referred for a pelvic CT scan, which, however, did not show any ovarian pathology. Due to an isolated persistent testosterone increase, we referred the patient for the performance of ovarian venous catheterization. This testing did not show clear laterization to one side but rather an extremely high bilateral testosterone production (Table 2) of ovarian origin, and the patient underwent bilateral oophorectomy. Histology showed the picture of ovarian stroma hyperthecosis without atypical cells/malignancy. Three months after surgery, the patient reported a reduction in her shave frequency, although she now complained about vasomotor symptoms due to rapid estrogen reduction after oophorectomy. Body weight was unaltered; however, the patient reported better blood glucose values, so that basal insulin units were reduced (from 14 to 8) and preprandial insulin was no longer necessary.

## 3. Case 2

A 74-year-old postmenopausal woman was referred to our endocrinology practice by her gynecologist due to increased total testosterone values. Patient's medical history revealed that she was in menopause since 15 years, and there were no prior indications for PCOS in the past. The patient was receiving hormone replacement therapy with estradiol/dihydrogesterone (1/15 mg) for 1 year, which was initiated because of progressive hair loss with a typical pattern for androgenetic alopecia, as well as acne along her front hairline. Furthermore, she developed excessive growth of thick hair in her upper lip and chin, which she removed every 2 days. Laboratory testing revealed increased total testosterone (370–400 ng/dL) and low adrenal androgen DHEA-S (Table 3). A 1 mg overnight dexamethasone suppression test showed formal MACS, which was probably a typical false positive result due to an increase in cortisol-binding globulin typically expected due to estradiol medication. Besides hirsutism, no other typical Cushing's signs were observed. Following discontinuation of hormone replacement therapy, estradiol remained detectable at a lower level, indicative of ongoing testosterone aromatization. MRI imaging of the abdominal and pelvic area did not show any adrenal or ovarian tumors; however, due to extremely high testosterone levels patient was referred for oophorectomy. From a gynecological standpoint, a total laparoscopic hysterectomy was performed. Histological examination of the right ovary revealed a 15 mm Leydig-cell tumor; otherwise, the right ovary did not show further alterations. The left ovary showed mild stromal hyperplasia without further abnormalities. The uterus demonstrated mild adenomyosis and, in focal areas, features consistent with early atypical endometrial hyperplasia, as well as multiple leiomyomas. Laboratory testing postoperatively showed decreased testosterone values. This was clinically evident by improvement of hirsutism and acne after the operation, without the need for further topical antiandrogenic therapy.

## 4. Case 3

We present the case of a 37-year-old patient who presented with a secondary amenorrhea for 7 years and a recent-onset hirsutism for the last 3–4 years. Hirsutism was evaluated using the gold-standard Ferriman–Gallwey score [12] (assessing terminal hair growth in 9 androgen-sensitive body areas and assigning a score from 0 to 4 for each area). Our patient had a Ferriman–Gallwey score > 16 and reported a daily shaving frequency. She reported a pronounced weight gain during her early adult years, with her highest body weight reaching 200 kg and the development of PCOS. Lifestyle modification led to a substantial weight loss (at the moment of presentation in our practice, she weighed 116 kg). Laboratory testing revealed an excessive isolated testosterone increase of 410 ng/dL in the presence of normal DHEA-S and androstenedione values and suppressed gonadotropins (Table 4). Anti-Müllerian hormone was initially increased (6 ng/mL). The patient attributed weakness and discomfort to the single administration of 1 mg dexamethasone and did not attend the scheduled morning blood sampling. Unfortunately, she refused to repeat the test. Overt hypercortisolism was excluded by normal 24-h cortisol urine extraction and intact circadian rhythm as suggested by the cortisol saliva test. The CT scan did not reveal adrenal or ovarian neoplasms. The patient was referred to a gynecological clinic for evaluation. Intravaginal ultrasound showed a well-defined hyperechoic solid mass within the left ovary without hypervascularization. In the right ovary, a small hypoechoic structure of 1.2 cm × 1.2 cm was revealed and interpreted as a follicle persistence. Further investigation included an MRI of the pelvic region, which confirmed the presence of the predescribed hyperintense mass (14 mm × 6 mm), whereas the right ovary did not show inhomogeneity. Due to the age of the patient, premenopausal status, and desire for pregnancy, caution was warranted as to specify diagnose and cautious treatment. For this reason, ovarian catheterization was also performed and showed increased production of testosterone in both ovaries with lateralization to the left ovary (left-to-right ratio of testosterone > 2.5, Table 5). The patient was referred for laparoscopy. A left-sided adnexectomy and, due to an intraoperatively suspicious ovarian tumor, a partial resection of the right ovary were performed. Histological examination showed the presence of a Leydig cell tumor on the left side and a serous borderline tumor of the right ovary (BOT). The tumors were completely resected (*R*0). Due to the desire for pregnancy and the presence of a borderline ovarian tumor, a fertility-sparing concept was attempted with the informed consent of the patient [13]. Due to high risk for gestational diabetes due to given factors (especially obesity and signs of PCOS), endocrinological follow-up was recommended, but unfortunately missed by the patient. She achieved pregnancy after ovarian stimulation therapy about 8 months after the surgery. The pregnancy was complicated by gestational diabetes, gestational hypertension, and cervical insufficiency. After receiving a cervical cerclage in the 22nd gestational week, the patient delivered following a cesarean section in the 32nd gestational week because of chorioamnionitis and preterm labor.

Three months after the delivery, she received a right-sided adnexectomy, an omentectomy, and peritoneal biopsies as completion surgery for BOT.

## 5. Case 4

**Figure 3:**
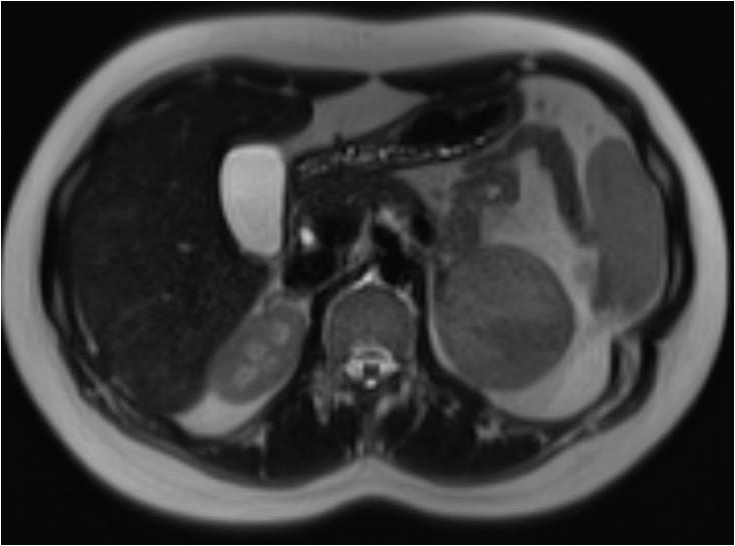
Abdominal CT scan demonstrating the androgen-producing adrenal tumor of the left adrenal gland in the patient of case 4.

Our last case is a 20-year-old female patient, who presented in our endocrinological practice with hirsutism (Ferriman–Gallwey score 8–12) for the last 3 years. The patient had a normal body weight with a BMI of 23 kg/m^2^. The hirsutism mainly affected the upper lip, chin, lower abdomen, and her back. She reported a regular menstrual cycle. Laboratory testing showed increased DHEA-S (1242 μg/dL) and testosterone values (Table 6). Due to high suspicion of an adrenal tumor, MRI imaging was promptly ordered and showed an adrenal adenoma of 60 mm × 55 mm in the left adrenal gland (Figure 3). Prolonged dexamethasone suppression test (0.5 mg dexamethasone every 6 h for 4 days) was performed and showed a lack of DHEA-S suppression, but an adequately suppressed morning cortisol, thus excluding ACS. Due to the size of the adrenal adenoma, but also high DHEA-S values, we referred our patient for left adrenalectomy. Histology confirmed an adrenal gland adenoma without suspicion of malignancy. After the operation, DHEA-S remained slightly elevated (400–600 μg/dL). Abdominal MRI did not show any residual tumor after the operation. The ACTH stimulation test did not indicate the presence of late-onset CAH. In this context, 17-hydroxypregnenolone was elevated in accordance with the DHEA-S level, suggesting reduced HSD3B2 activity. However, it did not exhibit the degree of stimulation typically expected in a genetic enzyme defect. Such a finding is frequently observed in cases of functional hyperandrogenism [14]. Clinically, our patients' hirsutism improved to a great extent after the operation. Further antiandrogenic therapies were not needed.

## 6. Discussion

In our first case, our patient presented with an adrenal adenoma and clinical signs of hypercortisolism as well as severe hyperandrogenism. Mild autonomous cortisol production was present along with a rapid onset of type 2 diabetes and an isolated testosterone increase, without a parallel increase of DHEA-S or androstenedione. However, adrenal androgen production from benign or (more frequently) malignant adrenal tumors is typically characterized by a parallel increase in serum testosterone and DHEA-S values, so in our case adrenal source of testosterone seemed unlikely. On the contrary, DHEA-S was in the lower normal range, and ACTH was below the normal range in accordance with adrenal autonomous cortisol production. 1 mg overnight dexamethasone testing is very helpful in these conditions, since suppression of testosterone within normal range can exclude tumorous processes (adrenal and ovarian) leading to hyperandrogenism [15]. Our patient already provided overnight 1 and 2 mg dexamethasone tests with only cortisol measurements, as performed by her house doctor, and was not willing to repeat testing due to distance and time reasons, so that we do not have this helpful information on testosterone suppression after dexamethasone. ACS is present in 20% of adrenal incidentalomas and is characterized by biochemical evidence of excessive cortisol production with or without typical signs of clinical hypercortisolism [16]. Due to signs of clinical hypercortisolism (striae rubriae, central adiposity, rapid onset of type 2 diabetes, arterial hypertension), she was referred for unilateral adrenalectomy. Since postoperative laboratory testing pointed to an ovarian androgen source, we suggested ovarian imaging before performance of adrenalectomy, which, however, did not reveal any abnormalities (e.g., increased ovarian volume), as previously described for ovarian hyperthecosis. Ovarian hyperthecosis is characterized by the presence of nests of luteinized theca cells in ovarian stroma and is a relatively rare cause of postmenopausal hyperandrogenism with a reported prevalence of around 9.3% [17]. Theca cells are responsible for androgen production in the ovaries, responding to luteinizing hormone (LH), which is converted to estrogens in granulosa cells. The most important differential diagnosis for ovarian hyperthecosis is PCOS, which is still the most frequent reason for postmenopausal hyperandrogenism. In a large registry, which included data from 1205 consecutively recruited pre and postmenopausal women with at least one increased androgen, testosterone increase was a common feature in all postmenopausal females with ovarian hyperthecosis, and isolated testosterone increase, as found also in our first case, was present in 4 out of 7 patients. On the other hand, postmenopausal PCOS was characterized by increased androstenedione values after menopause [17]. Although a cut-off of testosterone values > 150 ng/dL is reported as suggestive for this clinical entity, there are cases in which testosterone values did not reach this value, but ovarian histology revealed the typical hyperthecosis pattern [18]. In our case, testosterone values were indeed around 150 ng/dL. Although ovarian hyperthecosis is suggested to be an extreme form of PCOS, most women with PCOS do not develop hyperthecosis in menopause; most frequently only have less severe clinical manifestations, and their ovarian volume is normal, in contrast to enlarged ovaries in hyperthecosis [1]. In our case, however, increased ovarian volume was not confirmed by transvaginal ultrasound or CT imaging. Ultrasound findings are generally described as nonspecific, mainly limited to ovarian enlargement-although normal size does not exclude it, without focal areas of increased vascularization. MRI could be more helpful, showing bilateral ovarian enhancement with T2 hypodensity, but its role is also not clearly established [19]. Clinically, hyperthecosis becomes evident as slow-progress virilization (hirsutism, acne, androgenetic alopecia) in the presence of insulin resistance and metabolic syndrome [1]. Women with hyperthecosis more frequently exhibit insulin resistance as well as increased risk for type 2 diabetes mellitus and cardiovascular disease [20], which is not reversed after operation. Our patient experienced an improvement in her glucose metabolism after adrenalectomy, which continued after the next operation 1 year later. In this case, her glucose metabolism might have been affected by both endocrine abnormalities (hypercortisolism and hyperandrogenism). Further follow-up will show to what extent her metabolic profile profited from these interventions.

Ovarian hyperthecosis should also be differentially diagnosed from ovarian tumors. Here, careful patient history and clinical examination usually reveal more rapid-onset clinical features of hyperandrogenism, in the presence of more pronounced testosterone increases, as shown in our two patient cases with Leydig cell tumors. Ovarian catheterization can be a valuable tool in this context, providing or not lateralization of androgen production, which also has therapeutic consequences. In our first case, although ovarian hyperthecosis was discussed as the most likely diagnosis based on extremely high androgen release from both ovaries without clear laterization, oophorectomy was preferred over antiandrogen medication (spironolactone, GnRH analogs), in order to confirm the diagnosis. Androgen-secreting tumors are rare (about 1% of ovarian tumors) [21], predominantly benign, and account for around 2.7% of postmenopausal hyperandrogenism [17]. They include androblastomas (Sertoli, Sertoli–Leydig, and Leydig tumors), which typically secrete androgens (increased testosterone, but also androstenedione and 17-hydroxyprogesterone), thecomas and granulosa cell tumors, which are characterized primarily by estrogen secretion [22, 23]. Leydig cell tumors are very rare (< 0.1% of all ovarian tumors), often small, unilateral, and benign [24]. Due to their size, they may not always be identified by conventional imaging. In ultrasound, they may present isoechoic to myometrium, whereas MRI imaging may not be conclusive depending on their fibrous content [19]. In the case of our postmenopausal patient, pelvic MRI failed to identify it preoperatively, so clinical suspicion is key to further examinations. Furthermore, these tumors may coexist or develop on the grounds of obesity-related hyperandrogenism, which may make the right diagnosis more challenging and complicated, as in our case 3. PCOS diagnosis requires a combination of criteria (2 out of 3), including oligo/amenorrhea, polycystic ovarian morphology (PCOM), and presence of clinical/biochemical hyperandrogenism. Increased anti-Müllerian hormone values are indicative of this clinical entity and have lately gained attention as PCOS diagnosis criterion alternative to PCOM, although it is subject to certain limitations [25]. Genetic background also seems to play a role, since specific genetic polymorphisms have been associated with increased susceptibility to the syndrome [26]. In our case 3, high testosterone values, not typical for PCOS alone, called for further investigation. Normal DHEA-S values indicated an ovarian origin of hyperandrogenism, although increased DHEA-S values are also present in the context of PCOS [27] and may even be the most common laboratory constellation in premenopausal PCOS according to patient registry data [17]. As illustrated by our case 3, premenopausal status and desire for pregnancy can require special caution. Selective ovarian vein catheterization can be a helpful tool for this purpose, as previously described in females with hyperandrogenism, whose increased androgens were not adequately suppressed after a low-dose dexamethasone test, and ovarian imaging was inconclusive [28]. The present case was complicated by a second hormone-inactive tumor in the contralateral ovary, and a tissue-preserving operation was suitable for preserving fertility, proven by a pregnancy with delivery 15 months after removal of the left ovary and partial removal of the right ovary.

On the other hand, adrenal androgen-secreting tumors, although less common, even extremely rare [3] than ovarian tumors, are a possible differential diagnosis that needs to be ruled out. About 50% of these tumors are benign [29]. High DHEA-S and DHEA values can often help distinguish between ovarian and adrenal pathology. DHEA-S concentration is often above 700 μg/dL [1], androstenedione and testosterone are also increased. Furthermore, concomitant cortisol secretion and the presence of clinical features of Cushing's syndrome may be present. Computer tomography of adrenal glands, as in our case 4, usually reveals the presence of adenomas or carcinomas (usually larger than adenomas). Operation is the treatment of choice also for these tumors. Postoperative DHEA-S values serve as a marker for operation success. In our patient, persistent-although clearly less pronounced, increases of DHEA-S led us to further examinations to rule out, most importantly, residual tumor as well as underlying late-onset adrenogenital syndrome before confirming functional hyperandrogenism. Ultimately, the hyperandrogenism of this patient would be classified as predominantly resulting from an androgen-secreting adrenal tumor on the grounds of PCOS.

Longer-term follow-up examinations, including laboratory testing, are intended for our patients at annual intervals. These are important in order to document changes in virilization symptoms, identify possible disease recurrence at an early stage, but also to evaluate changes in glucometabolic control/insulin resistance, especially in the presence of an underlying PCOS.

**Figure 4:**
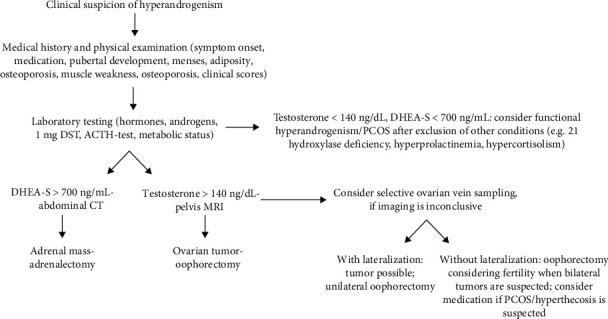
Proposed diagnostic approach of hyperandrogenism from clinical suspicion to laboratory testing, noninvasive imaging, and ovarian sampling.

Finally, upon clinical suspicion of hyperandrogenism, medical history, physical examination, and laboratory testing are required for correct diagnosis (Table 7). PCOS remains the predominant cause of hyperandrogenism in both pre and postmenopausal females and may be characterized by increased testosterone, androstenedione, and DHEA-S levels; however, rare causes of hyperandrogenism may be present or even coexist with PCOS. More pronounced increases in testosterone (>150 ng/dL) or DHEA-S (>700 μg/dL) should promptly lead to ovarian/abdominal imaging. We show that in some clinical entities (ovarian hyperthecosis, small ovarian tumors), imaging can be inconclusive; here, selective ovarian vein sampling can be a very valuable tool in order to confirm androgen hypersecretion and provide laterization, which can be important specifically in premenopausal females (Figure 4). Postoperative follow-up is important in order to identify possible underlying functional hyperandrogenism, evaluate remission of clinical symptoms, and metabolic risk factors often related to hyperandrogenism.

## 7. Conclusions

Tumorous etiology of hyperandrogenism can be present in pre and postmenopausal females and may coexist with more frequent causes of hyperandrogenism (e.g., PCOS). Medical history, clinical examination, and laboratory testing serve as a first-line approach; however, their diagnosis can require a combination of further examinations, for example, transvaginal ultrasound, magnetic resonance imaging (abdominal and pelvic), ovarian catherization, but also laparoscopy. Early recognition is of major importance in order to prevent permanent virilization and metabolic dysregulation.

## Figures and Tables

**Table 1:** Laboratory measurements of the patient in case 1 at first visit, after unilateral adrenalectomy due to adrenal adenoma, and after oophorectomy.

Parameters	Units	Normal range	First visit	After unilateral adrenalectomy	After oophorectomy
Serum cortisol	μg/dL	Morning (7 bis 10 Uh): 6–18.4	4.41	9.67	4.64
ACTH	pg/mL	7.2–63.3	5.67	41.6	22.0
Estradiol	pg/mL	35–104	30.9	24.7	<5.00
LH	IU/L	Postmen > 12	26.1	28.2	32.5
FSH	IU/L	Postmen > 12	34.0	32.1	42.9
LH/FSH	—	—	0.77	0.88	0.77
Total testosterone (ECLIA)	ng/dL	12–48	154	144	<2.50
SHBG	nmol/L	27.1–128	35.7	32.4	30.1
Free androgen index	kA	<4.5	14.9	14.7	n.a.
Androstenedione	ng/dl	49–131	77	100	<15
DHEA-S	μg/dL	19–205	22.0	12.0	12.3
17-Hydroxyprogesteron	ng/dL	11–108	128	167	25
HbA1c	%	4–5.69	7.8	5.7	5.5

**Table 2:** Testosterone, androstenedione, and estradiol values of selective ovarian catheterization of the patient in case 1.

Parameters	Units	Vena cava inferior	Left ovary	Right ovary	Right-to-left ratio	Left-to-right ratio
Estradiol	pg/mL	39.3	403	637	—	—
Total testosterone	ng/dL	134	5832	8800	1.5	0.7
Androstenedione	ng/dL	5.6	479	557	—	—

**Table 3:** Laboratory measurements of the patient in case 2 at first visit, after a pause of hormone replacement therapy (HRT), and after bilateral oophorectomy.

Parameters	Units	Normal range	First visit	After pause of HRT	After oophorectomy
Serum cortisol	μg/dL	Morning (7 bis 10 Uh): 6–18.4	12.8	—	—
ACTH	pg/mL	7.2–63.3	9.00	—	—
Estradiol	pg/mL	35–104	81.1	26.6	6.6
LH	IU/L	>12	6.1	7.2	12.9
FSH	IU/L	>12	9.5	14.9	23
LH/FSH	—	—	0.64	0.48	0.56
Total testosterone	ng/dL	12–48	373	380	2.55
SHBG	nmol/L	27.1–128	54.6	30.5	45.8
Free androgen index	—	<4.5	23.7	43.2	0.2
Androstenedione	ng/dL	49–131	127	107	55
DHEA-S	μg/dL	19–205	25.5	28.2	<15
17-hydroxyprogesterone	ng/dL	11–108	414	365	58

**Table 4:** Laboratory measurements of the patient in case 3 at the first visit and after the ovarian operation.

Parameters	Units	Normal range	First visit	After ovarian operation
Serum cortisol	μg/dL	Morning (7 bis 10 Uhr): 6–18.4	6.72	—
ACTH	pg/mL	7.2–63.3	13.3	—
Estradiol	pg/mL	Follicular phase 45–140	50.9	31
LH	IU/L	Follicular phase 1.5–3.0	0.7	8.4
FSH	IU/L	Follicular phase 1.8–3.5	0.9	8.4
LH/FSH	—	—	0.78	1
Total testosterone (ECLIA)	ng/dL	12–48	410	30
SHBG	nmol/L	27.1–128	21.6	23.1
Free andogen index	—	<4.5	65.8	4.5
Androstenedione	ng/dL	49–131	152	170
DHEA-S	μg/dL	19–205	126	158
17-hydroxyprogesterone	ng/dL	Follicular phase 20–100	163	—
Anti-Müllerian hormone	ng/mL	1.0–5.0	6.0	4.7

**Table 5:** Testosterone, androstenedione, and estradiol values of selective ovarian catheterization of the patient in case 3.

Parameters	Units	Vena cava inferior	Left ovary	Right ovary	Right-to-left ratio	Left-to-right ratio
Estradiol	pg/mL	86.8	682	142	—	—
Total testosterone	ng/dL	348	78,000	705	<0.47	110
Androstenedione	ng/dL	3.2	152	15.8	—	—

**Table 6:** Laboratory measurements of the patient in case 4 at the first visit and after unilateral adrenalectomy.

Parameters	Units	Normal range	First visit	After unilateral adrenalectomy
Serum cortisol	μg/dL	Morning (7 bis 10 Uh): 6–18.4	19.4	8.41
ACTH	pg/mL	7.2–63.3	33.0	12.7
Estradiol	pg/mL	Follicular phase 45–140	27.3	42.5
LH	IU/L	Follicular phase 1.5–3.0	1.6	1.8
FSH	IU/L	Follicular phase 1.8–3.5	1.6	2.3
LH/FSH	—	—	1	0.78
Total testosterone (ECLIA)	ng/dL	12–48	152	36.8
SHBG	nmol/L	27.1–128	18.3	35.5
Free androgen index	—	<4.5	28.8	3.6
Androstenedione	ng/dL	49–131	395	181
DHEA-S	μg/dL	19–205	2671	414
17-hydroxyprogesterone	ng/dL	20–100	210	108
Anti-Müllerian hormone	ng/mL	1.0–5.0	—	2.4

**Table 7:** Differential diagnosis of hyperandrogenism: prevalence and key clinical and laboratory features [3, 4, 17, 21, 27, 30–37].

Conditions	Prevalence/Incidence	Key clinical features	Key laboratory features
PCOS	5%–18% of women [30]. The most common cause of HA (up to 89% in premenopausal women) [17].	Onset during puberty, mild to severe hyperandrogenism, often related to obesity/insulin resistance.	Increased total testosterone (commonly < 100 ng/dL), androstenedione. Increased DHEA-S (~20%–30%) [26].
CAH/NCAH	Prevalence ∼1:14,000–1:18,000 [31].	Congenital, severe hyperandrogenism, virilization.	Increased 17-hydroxyprogesterone (CYP21A2-deficiency). Increased 11-deoxycortisol (CYP11B1-deficiency). Increased 17-pregnenolone, DHEA/DHEA-S (HSD3B2-deficiency). Increased ACTH, androstenedione. Increased renin in salt-wasting form.
Late-onset CAH	~1:1000 [32].	Phenotype mimicking PCOS.	Increased androgens, 17-hydroxyprogesterone.
Androgen-secreting ovarian tumor	~1% of ovarian tumors [33].	Severe hyperandrogenism, typically after puberty, more rapid onset.	Markedly increased testosterone (>150 ng/dL).
Androgen-secreting adrenal tumor	Pure androgen-secreting: <0.1% of adrenal tumors [3].	Severe hyperandrogenism, typically after puberty, more rapid onset. DHEA-S markedly increased; pure testosterone-secreting tumors are extremely rare.	Markedly increased DHEA-S (> ~700 μg/dL).
Ovarian hyperthecosis	9.3% of postmenopausal hyperandrogenism [17].	Typically, postmenopausal severe hyperandrogenism with slow progress, associated with obesity and metabolic syndrome.	Increased total testosterone (often > 100 ng/dL), androstenedione.
Secondary hyperandrogenism due to other endocrine diseases	—	Typical signs of specific hormone excess.	—
– Cushing-syndrome	1.8–4.5 cases/million/year [34]. PCOS findings in 46% of women with CS [4].	Specific: muscle weakness, striae rubrae, rubeosis faciei. Hyperandrogenism: mild to severe (more severe in ACTH-dependent form).	Increased cortisol, loss of diurnal variation. Failure of suppression in dexamethasone test. ACTH, suppressed or low-normal in ACTH-independent CS; in mid–high reference range in ACTH, dependent Cushing. Hypokalemia (esp. in ectopic CS). Hyperglycemia. Increased androgens (not mandatory).
– Acromegaly	0.2–1.1 cases/100,000/year [35]. PCOS findings in 33% of premenopausal women [36].	Specific: swelling of hands and feet, macroglossia, prominent forehead, and jaw.	Increased IGF-1, failure to suppress growth hormone levels during an oral glucose tolerance test.
– Hyperprolactinemia	3–5 cases/100,000/year. Prevalence 50/100,000 [37].	Amenorrhea and galactorrhea	Increased prolactin, hypogonadotropic hypogonadism. Occasionally increased DHEA-S [29].

## Data Availability

The data are available upon request from the authors.
